# The North Pole

**Published:** 1856-03

**Authors:** Jno Cleves Symmes

**Affiliations:** Ohio, late Captain of Infantry; St. Louis, (Missouri Territory,) North, America


### THE NORTH POLE.

Graham's Magazine for February, 1856, has a most interesting original article, being a sketch of Dr. Kane, who at the age of thirty-four years finds himself world-renowned, not as a human butcher, alias military chieftain, but celebrated for the humanity of his nature, for all that is noble, brave, daring, and all for the love of others, for the bare chance of saving from a miserable death, men whom he had never seen, but whom he considered his brethren in science and the sea. Having greatly impaired his constitution by exposures in southern latitudes, he seems to have greatly improved it by a succession of exposures to an atmosphere eighty degrees below zero. This is a fact worth noting by the multitudinous many who seek health by "going to the south."

We have been informed that Dr. Kane in personal appearance, action and tone of voice, is the twin of ourself. In one respect we know there is a striking difference, Dr. Kane, like all great men, is universally known as a man of great modesty: we do not really believe that we know what that is, we are principled against modesty, which is considered to be thinking less of one's self than is warranted. We cannot see the practical use of that. We are at all places and at all times of the Utilitarian School. We never could see the advantage of underestimating one's self. Certain are we, that as great violence is done to truth in undervaluing as in overvaluing. We have always taken the wise mean and considered the safest side of error to be, in the over-estimate. Every observant man must know that brass is durable, is sustaining; brazen-faced people go with a glory, where the mealy-mouthed would perish; and if the accumulation of money be the criterion of life-success, we can mention scores of individuals whose only element of success consisted in having consciences cast in brass. But we must here make a metaphysical distinction which is of great value. Self-reliance is the true metal, combined as it always is with indomitable energy. The opposite class of persons are what may be called the self-opinionated, who have the brass in sound, the brassy ring, without any of its enduring qualities.

Dr. Kane has the enviable distinction of having stood with his small and delicate frame, of five feet and a half, with less than a hundred and thirty pounds, a degree of cold, and at a higher elevation of latitude, than any other civilized man—he reached the point of eighty-two and a half degrees north latitude, within seven and a half degrees of the north pole, where he beheld a sea of unpenetrated ice eighty miles across, beyond which were evidences of land, and vegetation and animal life, with a temperature as promising as an Italian sky. What fountain of health there may be beyond that icy sea, and within that circle which no mortal has yet been known to enter, the future only can develope, but the following circular, written nearly forty years ago by one who was considered by some, as our friend his son now is, and perhaps as incorrectly, will be read with interest at this time. It was addressed:

" *To the town of Louisville, as a Body Corporate,* (*per mail,*) *Kentucky.*

LIGHT GIVES LIGHT, TO LIGHT DISCOVER—'AD INFINITUM.'

ST. LOUIS, (Missouri Territory,)
*North America, April* 10, *A. D.*, 1818.

TO ALL THE WORLD!

I declare that the earth is hollow, and habitable within; containing a number of solid concentrick spheres, one within the other, and that it is open at the poles 12 or 16 degrees; I pledge my life in support of this truth, and am ready to explore the hollow, if the world will support and aid me in the undertaking. JNO CLEVES SYMMES,
*Of Ohio, late Captain of Infantry.*

N. B.—I have already for the press a Treatise on the principles of matter, wherein I show proofs of the above positions, accounts for various phenomena, and disclose *Doctor Darwin's Golden Secret.*

My terms are the patronage of this and the new world.

I dedicate to my wife and her ten children.

I select *Doctor S. L. Mitchell*, *Sir H. Davy*, and *Baron Alex. de Humboldt*, as my protectors.

I ask one hundred brave companions, well equipped, to start from Siberia in the fall season, with Reindeer and slays, on the ice of the frozen sea; I engage we find warm and rich land, stocked with thrifty vegetables and animals if not men, on reaching one degree northward of latitude 82; we will return in the succeeding spring. J. C. S.

